# Supplementary material for: HIVprotI: an integrated web based platform for prediction and design of HIV proteins inhibitors
Source: J Cheminform. 2018 Mar 9;10:12. doi: 10.1186/s13321-018-0266-y (PMC5845081; doi:10.1186/s13321-018-0266-y)
Supplement: Supplementary file 2 — Additional file 2. Source code of HIVProtI web server. [file 13321_2018_266_MOESM2_ESM.zip › HIVprotI_Source-code/hivprotI/weka-3-6-6/doc/weka/gui/beans/package-frame.html]

weka.gui.beans


weka.gui.beans

Interfaces 
  
*BatchClassifierListener*
  
*BatchClustererListener*
  
*BeanCommon*
  
*ChartListener*
  
*ConnectionNotificationConsumer*
  
*CustomizerCloseRequester*
  
*CustomizerClosingListener*
  
*DataFormatListener*
  
*DataSink*
  
*DataSource*
  
*DataSourceListener*
  
*EventConstraints*
  
*GraphListener*
  
*IncrementalClassifierListener*
  
*InstanceListener*
  
*LogWriter*
  
*Startable*
  
*StartUpListener*
  
*StructureProducer*
  
*TestSetListener*
  
*TestSetProducer*
  
*TextListener*
  
*ThresholdDataListener*
  
*TrainingSetListener*
  
*TrainingSetProducer*
  
*UserRequestAcceptor*
  
*Visible*
  
*VisualizableErrorListener*
  
*WekaWrapper*

Classes 
  
AbstractDataSink
  
AbstractDataSinkBeanInfo
  
AbstractDataSource
  
AbstractDataSourceBeanInfo
  
AbstractEvaluator
  
AbstractTestSetProducer
  
AbstractTestSetProducerBeanInfo
  
AbstractTrainAndTestSetProducer
  
AbstractTrainAndTestSetProducerBeanInfo
  
AbstractTrainingSetProducer
  
AbstractTrainingSetProducerBeanInfo
  
Associator
  
AssociatorBeanInfo
  
AssociatorCustomizer
  
AttributeSummarizer
  
AttributeSummarizerBeanInfo
  
BatchClassifierEvent
  
BatchClustererEvent
  
BeanConnection
  
BeanInstance
  
BeanVisual
  
ChartEvent
  
ClassAssigner
  
ClassAssignerBeanInfo
  
ClassAssignerCustomizer
  
Classifier
  
ClassifierBeanInfo
  
ClassifierCustomizer
  
ClassifierPerformanceEvaluator
  
ClassifierPerformanceEvaluatorBeanInfo
  
ClassValuePicker
  
ClassValuePickerBeanInfo
  
ClassValuePickerCustomizer
  
Clusterer
  
ClustererBeanInfo
  
ClustererCustomizer
  
ClustererPerformanceEvaluator
  
ClustererPerformanceEvaluatorBeanInfo
  
CostBenefitAnalysis
  
CostBenefitAnalysisBeanInfo
  
CrossValidationFoldMaker
  
CrossValidationFoldMakerBeanInfo
  
CrossValidationFoldMakerCustomizer
  
DataSetEvent
  
DataVisualizer
  
DataVisualizerBeanInfo
  
Filter
  
FilterBeanInfo
  
FilterCustomizer
  
FlowRunner
  
FlowRunner.SimpleLogger
  
GraphEvent
  
GraphViewer
  
GraphViewerBeanInfo
  
IncrementalClassifierEvaluator
  
IncrementalClassifierEvaluatorBeanInfo
  
IncrementalClassifierEvaluatorCustomizer
  
IncrementalClassifierEvent
  
InstanceEvent
  
InstanceStreamToBatchMaker
  
InstanceStreamToBatchMakerBeanInfo
  
KnowledgeFlow
  
KnowledgeFlowApp
  
Loader
  
LoaderBeanInfo
  
LoaderCustomizer
  
LogPanel
  
Messages
  
MetaBean
  
ModelPerformanceChart
  
ModelPerformanceChartBeanInfo
  
PredictionAppender
  
PredictionAppenderBeanInfo
  
PredictionAppenderCustomizer
  
Saver
  
SaverBeanInfo
  
SaverCustomizer
  
ScatterPlotMatrix
  
ScatterPlotMatrixBeanInfo
  
SerializedModelSaver
  
SerializedModelSaverBeanInfo
  
SerializedModelSaverCustomizer
  
StripChart
  
StripChartBeanInfo
  
StripChartCustomizer
  
TestSetEvent
  
TestSetMaker
  
TestSetMakerBeanInfo
  
TextEvent
  
TextViewer
  
TextViewerBeanInfo
  
ThresholdDataEvent
  
TrainingSetEvent
  
TrainingSetMaker
  
TrainingSetMakerBeanInfo
  
TrainTestSplitMaker
  
TrainTestSplitMakerBeanInfo
  
TrainTestSplitMakerCustomizer
  
VisualizableErrorEvent
